# FAK-inhibition opens the door to checkpoint immunotherapy in Pancreatic Cancer

**DOI:** 10.1186/s40425-017-0217-6

**Published:** 2017-02-21

**Authors:** Stefan N. Symeonides, Stephen M. Anderton, Alan Serrels

**Affiliations:** 1 MRC Centre for Inflammation Research, The Queen’s Medical Research Institute, University of Edinburgh, Edinburgh, EH16 4TJ UK; 2 Edinburgh Cancer Research UK Centre, Institute of Genetics and Molecular Medicine, University of Edinburgh, Edinburgh, EH4 2XR UK

**Keywords:** FAK, Immunotherapy, Checkpoint blockade, Pancreatic cancer

## Abstract

Immunotherapy has had remarkable success in the treatment of some cancer types. However, pancreatic cancer has remained largely refractory to immunotherapy, including immune checkpoint inhibitors. Recently, Jiang and colleagues identified a key role for FAK in regulating the composition of the fibrotic and immuno-suppressive pancreatic tumour niche, and showed that FAK inhibitors can be used in combination with immune checkpoint blockade and gemcitabine chemotherapy to significantly delay pancreatic tumour progression. This study further supports the use of FAK inhibitors in combination with immunotherapy.

## Main Text

Despite our increased understanding of the pathology of pancreatic cancer, prognosis remains sadly limited. In the United Kingdom, 1-year survival for pancreatic adenocarcinoma presentations is just 21% and 5-year survival just 3% (Cancer Research UK) “(http://www.cancerresearchuk.org/health-professional/cancer-statistics/statistics-by-cancer-type/pancreatic-cancer)”. As a result, pancreatic cancer is the 4th to 5th most common cause of cancer death in the UK & US, and is predicted to be the second commonest cause by 2020. Improvements in combination chemotherapy for the fittest patients has come at the cost of significant toxicity, while novel agents have not yet made an impact. Harnessing the body’s own immune system to kill cancer cells has shown promise for a growing number of cancer types, most notably changing the treatment landscape of melanoma [1]. However, pancreatic cancer has remained largely refractory to immunotherapy, including immune checkpoint inhibitors [2]. In a recent issue of the journal *Nature Medicine*, Jiang and colleagues identify that inhibition of the non-receptor tyrosine kinase, Focal Adhesion Kinase (FAK), can sensitise genetically engineered mouse (GEM) models of pancreatic cancer to the anti-tumour effects of immune checkpoint blockade [3]. They show that this synergistic activity is underpinned by reprogramming of the fibrotic and immuno-suppressive pancreatic tumour microenvironment (TME) in response to treatment with a small molecule FAK kinase inhibitor, and that this is at least in part mediated through FAK-dependent regulation of chemokine expression. This work elegantly illustrates the potential for FAK kinase inhibitors to work alongside immunotherapy.

### FAK reprograms the immuno-suppressive pancreatic tumour niche

**Fig. 1:**
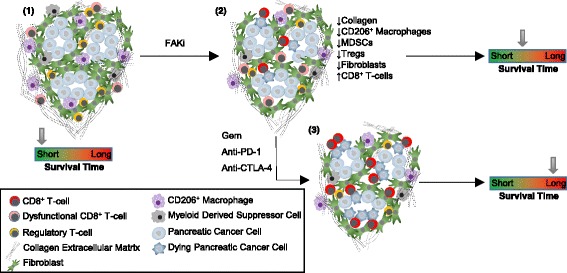
FAK inhibition reprograms the immuno-suppressive environment in pancreatic tumors rendering them sensitive to immune checkpoint blockade. (1) Pancreatic Ductal Adenocarcinoma contains a highly fibrotic and immuno-suppressive microenvironment. (2) Inhibition of FAK kinase activity results in extensive remodeling of the fibrotic and immuno-suppressive tumor niche that results in a modest improvement in survival time. (3) Combining the effects of FAK inhibition with checkpoint blockade and gemcitabine chemotherapy results in a significant improvement in survival time and even long-term survival

FAK is frequently upregulated in many cancer types, and signals downstream of integrins and growth factor receptors to control a variety of cellular functions that are important for the malignant phenotype, including adhesion, migration, invasion, proliferation, and survival [4]. Recently, we identified an unexpected role for FAK in regulating the anti-tumour immune response in a mouse model of skin squamous cell carcinoma (SCC) [5]. We found that FAK-dependent regulation of chemokines and cytokines in cancer cells was required to drive elevated levels of regulatory T-cells (Tregs) into the tumour environment, resulting in suppression of the anti-tumour CD8^+^ T-cell response. This was mediated through nuclear FAK signalling in cancer cells, and treatment with a small molecule FAK kinase inhibitor resulted in immune-mediated tumour regression. Thus, targeting FAK could unlock the anti-tumour immune response through regulating the immuno-suppressive tumour environment. Jiang and colleagues investigated the immuno-modulatory effects of FAK kinase inhibition in GEM models of pancreatic cancer, and explored the potential for combination with immune checkpoint blockade [3]. They identified that FAK signalling in cancer cells acts to broadly regulate the composition of the TME. They report a reduction in the number of tumour infiltrating (1) FAP^+^ fibroblasts, (2) myeloid derived suppressor cells (MDSC), (3) CD206^+^ macrophages, and (4) Tregs (Fig. 1). In some instances, this was also accompanied by elevated CD8^+^ T-cell infiltration into FAK-depleted tumours. In addition, the authors also identified that FAK inhibition reduces collagen deposition within the tumour environment, overcoming a physical barrier to T-cell infiltration. These changes collectively reprogram the pancreatic TME, shifting the balance in favour of anti-tumour immunity. In seeking to explore the mechanism underpinning this, the authors identified that FAK regulates the expression/secretion of a number of chemokines in pancreatic cancer cells, consistent with previous reports using other cell types [5, 6]. In particular, they focused on CXCL12, demonstrating that it may play an important role in stromal expansion within the pancreatic tumour niche through driving increased proliferation of pancreatic fibroblasts. This observation is consistent with previous reports identifying a reduction in fibroblast infiltration into pancreatic tumours following treatment with a FAK inhibitor [7], and builds on our previous finding that FAK-dependent expression of the chemokine CCL5 in cancer cells was required to drive elevated levels of Tregs into SCC tumours [5]. Together, these findings identify that FAK-regulated chemokine expression is an important mechanism through which cancer cells exert influence over the composition of the surrounding TME in order to evade the anti-tumour immune response. Further work will be required to fully understand the mechanisms of FAK-dependent chemokine regulation, and its contribution to regulation of the TME in cancer.

### FAK inhibition synergises with immune checkpoint blockade to improve survival time

In seeking to explore the therapeutic value of FAK-dependent changes in the immuno-suppressive tumour environment, Jiang and colleagues made excellent use of the KPC and KPPC GEM models of pancreatic cancer. These tumours did not respond to the combination of anti-CTLA-4 and anti-PD-1, reflecting the outcomes of clinical trials [2]. However, combination with VS-4718 [8], one of a number of FAK kinase inhibitors in clinical development [9], and gemcitabine, one of the standard of care chemotherapies for pancreatic cancer, led to a synergistic and significant anti-tumour response and even long-term survival of a small number of mice from the cohort receiving this treatment (Fig. 1). These results are extremely encouraging and further support the rationale for FAK/immunotherapy combinations to be tested in the clinic. Furthermore, they provide additional evidence that immunotherapy may yet work in pancreatic cancer, if the right combination of agents can be found. However, clinical translation of this 4-way combination may require to be staged in order to fully understand the contribution of each agent and the potential toxicity each will add. For example, it is already established that the combination of anti-CTLA-4 and anti-PD-1 can result in potentially severe immune-mediated side-effects that require careful clinical management [1], prompting exploration of de-escalated dosing and/or scheduling. Clinical trials combining anti-PD-1 with gemcitabine are already underway, and similar trials incorporating FAK inhibitors have either started or are due to start soon. This is the first time FAK inhibitors have been tested in combination with immunotherapy and the preclinical data are very encouraging. However, we do not yet know if this is the optimal combination. Future studies will need to address this in detail, both in terms of efficacy and tolerability, for translation to the clinic.

### Clinical impact

The work reported by Jiang and colleagues has seen rapid clinical translation, and has resulted in a clinical trial to test the safety, tolerability, and anti-tumour efficacy of the FAK inhibitor defactinib in combination with pembrolizumab (anti-PD-1) and gemcitabine in advanced pancreatic cancer (clinicalTrials.gov NCT02546531). This will run alongside a further clinical trial that stemmed from our work, which will aim to assess the safety, tolerability, and anti-tumour efficacy of defactinib in combination with pembrolizumab in pancreatic cancer, non-small cell lung cancer, and mesothelioma (clinicalTrials.gov NCT02758587). With clinical testing now underway, we look forward to determining whether the highly encouraging pre-clinical data will translate into clinical benefit in treating these cancers of high unmet need. A key aspect of this ongoing work is the loop back to the laboratory, exploring those real-life biological effects, thereby refining and optimising future combination immunotherapy.

## Abbreviations

anti-CTLA4: Anti- cytotoxic T-Lymphocyte associated protein 4
anti-PD-1: Anti-programmed death receptor 1
FAK: Focal adhesion kinase
FAP: Fibroblast activation protein
GEM: Genetically engineered mouse
MDSC: Myeloid derived suppressor cells
SCC: Squamous cell carcinoma
TME: Tumour microenvironment
Tregs: Regulatory T-cells
